# Measuring Fluid Intelligence in Healthy Older Adults

**DOI:** 10.1155/2017/8514582

**Published:** 2017-01-30

**Authors:** Mohammed K. Shakeel, Vina M. Goghari

**Affiliations:** ^1^Department of Psychology, University of Calgary, 2500 University Drive NW, Calgary, AB, Canada T2N 1N4; ^2^Department of Psychology, University of Toronto, 1265 Military Trail, Toronto, ON, Canada M1C 1A4

## Abstract

The present study evaluated subjective and objective cognitive measures as predictors of fluid intelligence in healthy older adults. We hypothesized that objective cognitive measures would predict fluid intelligence to a greater degree than self-reported cognitive functioning. Ninety-three healthy older (>65 years old) community-dwelling adults participated. Raven's Advanced Progressive Matrices (RAPM) were used to measure fluid intelligence, Digit Span Sequencing (DSS) was used to measure working memory, Trail Making Test (TMT) was used to measure cognitive flexibility, Design Fluency Test (DFT) was used to measure creativity, and Tower Test (TT) was used to measure planning. The Cognitive Failures Questionnaire (CFQ) was used to measure subjective perceptions of cognitive functioning. RAPM was correlated with DSS, TT, and DFT. When CFQ was the only predictor, the regression model predicting fluid intelligence was not significant. When DSS, TMT, DFT, and TT were included in the model, there was a significant change in the model and the final model was also significant, with DFT as the only significant predictor. The model accounted for approximately 20% of the variability in fluid intelligence. Our findings suggest that the most reliable means of assessing fluid intelligence is to assess it directly.

## 1. Introduction

There has been a lot of interest in recent years in the use of subjective reports of cognitive functioning to predict the likelihood that a person will suffer from dementia [1]. However, the findings have been equivocal [2]. This may be in part because people are poor judges of their own mental functions [3], and this may be especially true of those who already have poor cognitive functioning [4]. This view has important implications for the use of subjective reports in dementias, which are characterized by cognitive, as well as metacognitive, deficits [5, 6]. There is a lack of empirical studies on the usefulness of making inferences about complex mental functions by assessing other, related mental functions (subjectively or objectively). This also has important implications for diagnosis, because it is often not feasible to administer time-consuming tests for complex functions (like intelligence) in a clinical setting, and may also require specific training in administration and interpretation. In contrast, though widely used, research has demonstrated that asking patients about their own mental functions may not be a fruitful approach [7, 8].

In the present study, we aimed to investigate, in healthy older adults, whether subjective and objective measures predicted complex cognitive functioning, namely, fluid intelligence. Fluid intelligence involves reasoning and problem solving for problems to which familiar solutions are not available. Fluid intelligence tends to decline with age [9] and is impaired in dementia [10]. Hence, fluid intelligence serves as an ideal candidate to investigate the usefulness of subjective and objective predictors for a complex cognitive function. However, as with many complex functions, it is not possible to reliably assess fluid intelligence using subjective reports. Therefore, we relied on using the Cognitive Failures Questionnaire, which requires the participant to answer questions that assess memory [11], attention [11], and executive functioning, which are related to fluid intelligence [12–14].

We also wanted to assess how well subjective reports of cognitive functioning perform compared to objective measures like working memory [15], cognitive flexibility [16], creativity [17], and planning [18], which are related to fluid intelligence [16, 19–22]. However, there is no consensus on the strength of the association between fluid intelligence and these variables (see, e.g., [19, 20, 23–25]), and it also remains to be determined whether the association holds for older adults as it does for other age groups.

Furthermore, for this study, we specifically chose objective measures that are neither time-consuming nor difficult to administer in a clinical setting. To avoid redundancy, we also ensured that the subjective and objective measures assessed nonoverlapping constructs related to fluid intelligence. We hypothesized that objective measures of related cognitive processes would predict fluid intelligence better than subjective report of cognition.

## 2. Materials and Methods

### 2.1. Participants

Sociodemographic information is presented in Table 1. The sample consisted of healthy older community-dwelling adults aged between 65 and 86 years, recruited from Calgary, Alberta. All participants were proficient in English. To reduce the effects of other factors that could affect cognitive functions or the ability to adequately perform the tasks, participants were excluded if they had a head trauma, brain fever, neurological illness, dementia or altered consciousness, history of recent (3 months) use of benzodiazepines or illicit drugs, current visual, auditory, or motor impairment, cardiovascular conditions, breathing problems or pathologies associated with cognitive impairment such as stroke, Parkinson's disease, intracranial hemorrhage, tumors, and normal pressure hydrocephalus, or a score of less than 27 on the Mini Mental State Examination [26]. Informed written consent was obtained from all participants. The study was approved by the University of Calgary Conjoint Faculties Research Ethics Board and is in accordance with the ethical standards of the Helsinki Declaration [27].

### 2.2. Measures

All data was collected in a single session held at the University of Calgary.

#### 2.2.1. Raven's Advanced Progressive Matrices (RAPM) [28]

Raven's Advanced Progressive Matrices were used as a measure of fluid intelligence. The task requires participants to examine a series of images and select one out of 8 possible images to complete the pattern. The test has 36 items of progressively increasing difficulty. The total correct score obtained was used for the present analysis.

#### 2.2.2. Digit Span Sequencing (DSS)

Digit Span Sequencing was used to measure working memory [29]. The test is part of the Wechsler Adult Intelligence Scale-IV (WAIS-IV). In this task, the participant has to mentally rearrange a series of verbally presented digits and recall them in sequential order. The total raw score was used for the present analysis.

#### 2.2.3. Trail Making Test (TMT)

Trail Making Test Condition 4 was used as a measure of cognitive flexibility. Trail Making Test is part of the Delis-Kaplan Executive Function System (D-KEFS) [30]. For this task, the participant has to connect circles as quickly as possible while sequentially switching between circles which contain numbers and letters. The total raw score was used for the present analysis.

#### 2.2.4. Design Fluency Test (DFT)

Design Fluency Test Condition 3 was used as a measure of creativity. It also measures problem solving ability and inhibition. It is part of the D-KEFS [30]. In this task the participant is presented with squares containing 10 dots, 5 empty and 5 filled in. The participant has to draw designs by connecting dots in the square while constantly switching between empty and filled dots. The participant is also asked to generate unique designs for each square without repeating any of the designs. The total raw score was used for the present analysis.

#### 2.2.5. Tower Test (TT)

Tower Test was used as a measure of planning. Tower Test is part of the D-KEFS [30]. In this test, the participant has to move 5 colored disks of different sizes across 3 pegs to match a position shown by the investigator. The participant is asked to complete the task with as few moves as possible, moving only one disk at a time and without placing a larger disk over a smaller disk. The task gets progressively more difficult as the trials increase. Completing the trial in fewer moves results in higher achievement scores (as long as the task is completed within the time limit; for details, see [30]). The total achievement score was used for the present analysis.

#### 2.2.6. Cognitive Failures Questionnaire (CFQ) [31]

Subjective evaluation of cognitive function in everyday life was assessed using the Cognitive Failures Questionnaire. Cognitive Failures Questionnaire is a 25-item self-report measure that evaluates difficulties in attention, memory, distractibility, and executive functions. The questionnaire has good validity and reliability [31]. An analysis of data from the Royal Navy showed that Cognitive Failures Questionnaire scores are also correlated with real world outcomes like accident proneness, human error, and psychological strain [32]. The participant has to read sentences and indicate how often in the past 6 months they have had any of the mentioned experiences. Examples of questions are “Do you leave important letters unanswered for days?”; “Do you fail to see what you want in a supermarket (although it is there)?”; “Do you start doing one thing at home and get distracted into doing something else (unintentionally)?”; “Do you say something and realize afterwards that it might be taken as insulting?”; “Do you find you cannot think of anything to say?”; and so forth. Higher scores on Cognitive Failures Questionnaire indicate more problems. The test showed good internal consistency in our sample (Spearman-Brown Coefficient for split-half reliability was .91). The total score was used for the present analysis.

### 2.3. Statistical Analysis

Complete data was available for 92 participants. The data was analyzed using correlational and hierarchical multiple regression analyses. Bayesian analysis was additionally used to evaluate the correlation between Cognitive Failures Questionnaire and Raven's Advanced Progressive Matrices. Five univariate outliers (with *z*-scores > 3.3) were excluded from the variables Cognitive Failures Questionnaire, Digit Span Sequencing, Design Fluency Test, and Trail Making Test. The final sample for analysis consisted of 93 participants. All assumptions for linear regression analysis were met.

## 3. Results 

To observe the interrelationships between variables, Pearson's bivariate correlation analysis was conducted. Raven's Advanced Progressive Matrices were significantly correlated with Digit Span Sequencing (*r*(91) = .22, *p* = .03; *t*(91) = 2.15, *p* = .03), Trail Making Test (*r*(91) = −.30, *p* < .003; *t*(91) = −3.0, *p* = .003), and Design Fluency Test (*r*(91) = .39, *p* < .01; *t*(91) = 4.04, *p* < .001), but not with Cognitive Failures Questionnaire or Tower Test. Digit Span Sequencing was significantly correlated with Trail Making Test (*r*(91) = −.24, *p* = .02; *t*(91) = −2.36, *p* = .02) and Tower Test (*r*(90) = .27, *p* = .01; *t*(91) = 2.66, *p* = .009). Trail Making Test was significantly correlated with Design Fluency Test (*r*(91) = −.41, *p* < .001; *t*(91) = −4.29, *p* < .001) and Tower Test (*r*(90) = −.21, *p* = .04; *t*(90) = −2.04, *p* = .04). Design Fluency Test was significantly correlated with Tower Test (*r*(90) = .31, *p* = .003; *t*(90) = 3.10, *p* = .002) (Tables 2 and 3).

Cognitive Failures Questionnaire was not correlated with fluid intelligence (*r*(91) = −.08, *p* = .45; *t*(91) = −0.76, *p* = .45) or any other measure. The correlation between Cognitive Failures Questionnaire and fluid intelligence was also examined by estimating a Bayes factor. The results showed that the correlation had a JZS BF_10_ of 0.19. This demonstrates that the data was over 5 times more likely to occur under a model where Cognitive Failures Questionnaire was not related to Raven's Advanced Progressive Matrices, rather than a model in which they were related. No other significant correlations were present.

### 3.1. Regressions

Means and standard deviations for all variables are presented in Table 4. Hierarchical linear regression with Raven's Advanced Progressive Matrices as the dependent variable and Cognitive Failures Questionnaire as the independent variable in block 1 showed that the Cognitive Failures Questionnaire did not significantly predict the score on Raven's Advanced Progressive Matrices (*F*(1, 90) = 0.49, *p* = .49). When Digit Span Sequencing, Trail Making Test, Design Fluency Test, and Tower Test were included in block 2, it resulted in a significant final model (*F*(5, 86) = 4.48, *p* = .001) which accounted for over 20% of the variability in Raven's Advanced Progressive Matrices (*R*^2^ = .21; adjusted *R*^2^ = .16). However, the only significant predictor in the final model was the Design Fluency Test (*B* = 0.33, *β* = .29, *t*(86) = 2.68, *p* = .009, and *pr*^2^ = .28) (Table 5).

A follow-up exploratory regression analysis was conducted to determine whether age was a potential contributing factor. Hierarchical regression analysis with Raven's Advanced Progressive Matrices as the dependent variable and participant's age as the independent variable in block 1 showed that age did not significantly predict the score on Raven's Advanced Progressive Matrices (*F*(1, 90) = 3.18, *p* = .78). When the cognitive variables (Cognitive Failures Questionnaire, Digit Span Sequencing, Trail Making Test, Design Fluency Test, and Tower Test) were included in block 2, the results remained similar to the first regression analysis with the cognitive variables resulting in a significant final model (*F*(6, 85) = 3.78, *p* = .002) of Raven's Advanced Progressive Matrices.

## 4. Discussion

These results demonstrated that measures of working memory, cognitive flexibility, and creativity were significantly associated with fluid intelligence, but planning and subjective report of cognitive functioning were not. We also found that creativity was the only significant predictor of fluid intelligence in the regression model, which included subjective report of cognitive functioning, as well as working memory, cognitive flexibility, and planning as predictors.

Our finding that subjective reports are poor predictors of objective cognitive functioning is in agreement with previous studies. For instance, one meta-analysis [2] reported that, in cross-sectional community settings, people who report subjective memory impairments only have a 20% chance of actually suffering from dementia, while 60% of people with dementia do not report any memory problems. This discrepancy between subjective judgements and the actual status of cognitive functions has also been demonstrated in basic cognition literature [3, 4]. Our findings extend this view to inferring the level of fluid intelligence based on subjective report of cognitive functions like attention, memory, distractibility, and executive functions. Subjective report of cognitive functioning was not associated with any of the objective measures either. This may be because the Cognitive Failures Questionnaire primarily assesses memory, attention, and executive functions, while the objective measures assess working memory (DSS), cognitive flexibility (TMT), creativity (DFT), and planning (TT), and there may be little overlap in the cognitive functions that the subjective and objective measures assess.

Our results showed that objective measures of cognitive functioning (working memory, cognitive flexibility, and creativity) are significantly associated with fluid intelligence. However, the final regression model only accounted for around 20% of the variability in fluid intelligence, and the test of creativity was the only significant predictor in the final model. By most standards, a linear regression model accounting for 20% variability is acceptable; however, the purpose of the present analysis was to determine whether the predictors could be used as substitutes for assessing fluid intelligence directly, hence saving time and effort in clinical settings. With that goal in mind, five tasks accounting for 20% of the variability are unfortunately not practically useful. Moreover, the test of creativity was the only significant predictor.

There is no consensus in the literature on the nature of the relationship between creativity and fluid intelligence; while some studies have found them to be strongly related [19, 20], others have only reported a weak association [23] or no relation [24]. Our findings provide partial support to the view that creativity and fluid intelligence are positively related. However, it must be noted that creativity only made a modest contribution to predicting fluid intelligence in our analysis and hence further studies are required to fully understand the strength and nature of the relation between these constructs.

There have been studies that have shown fluid intelligence to be associated with working memory [21], cognitive flexibility [16], and planning [22], although the strength of the association between fluid intelligence and other cognitive functions has been disputed (see, e.g., [25]). Unlike studies with mostly younger participants, we did not find working memory, cognitive flexibility, or planning to be significant individual predictors of fluid intelligence in our sample of healthy older adults. However, we did find an overall combined association of these variables with fluid intelligence.

One limitation of our study was including only a single self-report measure while multiple objective measures were used. Self-report measures of cognitive functioning in everyday life tend to assess several cognitive domains simultaneously and there is considerable overlap of constructs assessed by the Cognitive Failures Questionnaire with constructs assessed by other measures like Perceived Deficits Questionnaire [33], Patient-Reported Outcomes in Cognitive Impairment [34], and so forth. Hence, the use of multiple self-report measures of cognition in everyday life would be redundant. To control for multiple comparisons, individual predictors were analyzed only in the presence of a significant final model. However, we acknowledge that the use of a single self-report measure is a limitation of this study.

## 5. Conclusion

The aim of the present study was to investigate whether subjective or objective measures that are associated with fluid intelligence can be used as substitutes to measuring fluid intelligence directly. Given that subjective reports were not able to predict fluid intelligence and objective measures did not substantially account for fluid intelligence, we conclude that neither subjective nor objective measures used in this study can be used as substitutes to measuring fluid intelligence directly, at least in older adults. This does not rule out the possibility that future studies may identify other subjective/objective measures that can be reliably used as proxy measures, but our findings suggest that the only reliable way to assess complex cognitive functions is to assess them directly. We further recommend the use of the shorter version of Raven's Advanced Progressive Matrices, which takes around half the time as the full version yet adequately predicts scores on the full version [35].

## Figures and Tables

**Table 1:** Sociodemographic information (*n* = 93).

	Mean (standard deviation) or percentage
Age	70.12 (4.61)
Handedness (% right)	84.9
Sex (% female)	65.6
Ethnicity	
Caucasian (%)	89.2
Asian (%)	8.6
Others (%)	2.2
Education (years)	15.79 (3.51)
Employment status	
Retired (%)	83.9
Full time employed (%)	3.3
Part time employed (%)	8.7
Others (%)	4.4

**Table 2:** Correlations among variables (*n* = 93).

	RAPM	CFQ	DSS	TMT	DFT
RAPM					
CFQ	−.08				
DSS	.22^*∗*^	.06			
TMT	−.30^*∗∗*^	−.08	−.24^*∗*^		
DFT	.39^*∗∗*^	−.01	.20	−.41^*∗∗*^	
TT	.18	.01	.27^*∗∗*^	−.21^*∗*^	.31^*∗∗*^

RAPM = Raven's Advanced Progressive Matrices; CFQ = Cognitive Failures Questionnaire; DSS = Digit Span Sequencing; TMT = Trail Making test; DFT = Design Fluency Test; TT = Tower Test.

^*∗*^ *p* < .05.

^*∗∗*^ *p* < .01.

**Table 3:** Covariance among variables (*n* = 92).

	RAPM	CFQ	DSS	TMT	DFT	TT
RAPM	7.29					
CFQ	−2.84	205.38				
DSS	1.24	1.50	3.81			
TMT	−22.91	−30.70	−12.70	746.49		
DFT	2.53	−0.41	0.95	−26.69	5.72	
TT	1.94	0.86	2.13	−23.29	3.00	16.40

RAPM = Raven's Advanced Progressive Matrices; CFQ = Cognitive Failures Questionnaire; DSS = Digit Span Sequencing; TMT = Trail Making test; DFT = Design Fluency Test; TT = Tower Test.

**Table 4:** Mean (standard deviation) of measures (*n* = 93).

	Mean (standard deviation)
RAPM	8.02 (2.69)
CFQ	35.37 (14.29)
DSS	9.16 (1.95)
TMT	86.90 (27.22)
DFT	7.59 (2.38)
TT	16.65 (4.05)

RAPM = Raven's Advanced Progressive Matrices; CFQ = Cognitive Failures Questionnaire; DSS = Digit Span Sequencing; TMT = Trail Making test; DFT = Design Fluency Test; TT = Tower Test.

**Table 5:** Hierarchical regression analysis of Digit Span Sequencing, Trail Making Test, Design Fluency Test, and Tower Test on Raven's Advanced Progressive Matrices after controlling for Cognitive Failures Questionnaire (*n* = 92).

	*B*	SE	*β*	*pr* ^2^
Block 1				
Intercept	8.53	0.75		
CFQ	−0.01	0.02	−.07	−.07
*R*	.07			
Block 2				
Intercept	5.62	2.26		
CFQ	−0.02	0.02	−.09	−.10
DSS	0.19	0.14	.14	.14
TMT	−0.02	0.01	−.16	−.16
DFT	0.33	0.12	.29^*∗∗*^	.28
TT	0.01	0.07	.02	.02
*R*	.45			
*R* ^2^	.21			

RAPM = Raven's Advanced Progressive Matrices; CFQ = Cognitive Failures Questionnaire; DSS = Digit Span Sequencing; TMT = Trail Making test; DFT = Design Fluency Test; TT = Tower Test; *B* = *B*-value; SE = Standard Error; *β* = Beta; *pr*^2^ = partial *r*^2^.

^*∗∗*^ *p* < .01.
